# A low-cost open-source 3D-printed mouse cradle suspension system for awake or anaesthetised ${}^{1}$H/^31^P magnetic resonance spectroscopy

**DOI:** 10.1016/j.ohx.2024.e00616

**Published:** 2024-12-30

**Authors:** Saba Molhemi, Leif Østergaard, Brian Hansen

**Affiliations:** Institute of Clinical Medicine - Aarhus University, Center of Functionally Integrative Neuroscience (CFIN), Palle Juul Jensens Boulevard 99, Krydspunkt J117, 8200, Aarhus N, Denmark

**Keywords:** Phosphorus magnetic resonance spectroscopy, Proton magnetic resonance spectroscopy, Preclinical, Awake mouse, 3D printing, Computer-aided design

## Abstract

Awake mouse MRI and spectroscopy (MRS) are valuable techniques for studying biological questions without the confounding effects of anaesthesia. Currently, no off-the-shelf solution exists for awake mouse MRI/S. To address this, we present a Mouse Cradle Suspension System (MCSS) for awake mouse MRI/S. Our design is freely available and offers a low-cost 3D-printed setup compatible with a Bruker Biospec 94/20 scanner and commercially available ${}^{1}$H/^31^P surface- and volume-coils, such as coils from Bruker Biospin (T20025V3) and Rapid (O-XL-HL-094). While the focus here is measurements in awake mouse brain, the coils and the presented setup is suitable for both mouse and rat brain, and studies of mouse body organs. Moreover, the design is easily modifiable to suit other applications and hardware configurations. The MCSS reduces gradient-induced coil vibrations and supports cross-coil setups. It features an inner and outer rail system for easy insertion of the coil and customized mouse cradle into the scanner. The cradle is suitable for both anaesthetized and awake mouse scans and existing habituation protocols for awake mouse MRI/S. This MCSS design ensures a smooth workflow for awake mouse MRI/S. The cost is approximately 200€, achieved using 3D-printed and off-the-shelf components.

## Graphical abstract

**Figure:**
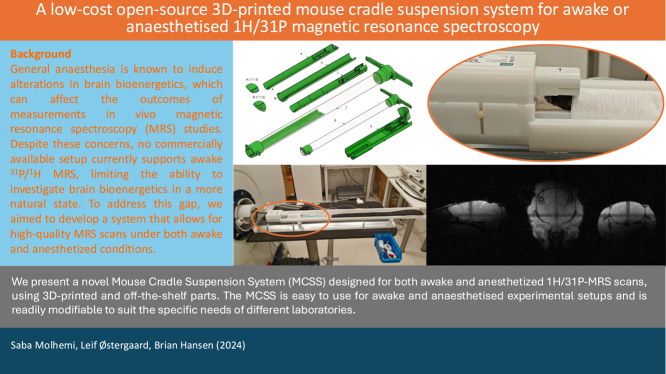

## Specifications table

**Table:** 

Hardware name	Mouse Cradle Suspension System (MCSS)
Subject area	•Mouse proton magnetic resonance spectroscopy • Mouse phosphorus magnetic resonance spectroscopy • Neuroscience • In vivo spectroscopy • Preclinical MRS

Hardware type	•Setup for awake and anaesthetized mouse ${}^{1}$H/${}^{31}$P MRI/S • Setup for awake mouse whisker stimulation ${}^{1}$H/${}^{31}$P MRI/S • Setup for anaesthetized mouse ${}^{1}$H/${}^{31}$P MRI/S

Closest commercial analog	No commercial analog is available.

Open source license	CC BY 4.0

Cost of hardware	app. 200€

Source file repository	https://doi.org/10.17632/yn9ydywcby.1

## 1 Hardware in context

Phosphorous magnetic resonance spectroscopy (MRS) holds promise as a tool for non-invasive investigation of body energetics. Such tools are particularly important for brain studies. The brain has high energy consumption but little to no energy storage. Therefore, constant energy supply and turnover is crucial for maintaining normal brain function and health. However, this process is often disrupted in pathological conditions like traumatic brain injury (TBI) [1], brain tumor [2], [3], and stroke [4], [5], [6]. Additionally, in neurodegenerative diseases (NDD) such as Alzheimer’s disease (AD) [7], [8] and Parkinson’s disease (PD) [9], subtle alterations in energy metabolism precede cognitive decline. NDDs have been combated for decades with little success in the most common diseases. Today the general strategy for combating NDDs is to understand early mechanisms driving the pathology for early detection and prevention [10]. The metabolic function in NDDs is therefore receiving increased attention. Consequently, tools capable of detecting energy metabolism in normal and diseased states are important. In vivo phosphorous MRS is one such promising tool. MRS provides a chemical spectrum of metabolites present in a defined volume of interest (VOI). Most in vivo applications utilize proton (^1^H) MRS which has the highest MR sensitivity owing to its high gyromagnetic ratio and high natural abundance (≥%99.9) with most in vivo metabolites containing ^1^H. (^1^H) MRS allows the detection of important amino acids, such as γ-Aminobutyric Acid (GABA), glutamate, glutamine, N-Acetyl Aspartate (NAA), aspartate and the byproduct of glycolysis, lactate [11]. ^31^P-MRS has an MR sensitivity of 6.7% compared to ^1^H, but gives metabolic insight not available by ^1^H-MRS [12], [13]. In vivo ^31^P-MRS is capable of detecting two major classes of metabolic markers in a defined VOI, that is, metabolites linked to bioenergetic metabolism and phospholipid membrane turnover. These bioenergetic metabolites are adenosine triphosphate (ATP), phosphocreatine (PCr) and inorganic phosphate (Pi). Phosphomonoesters (PMEs) are crucial precursors in the synthesis of membrane phospholipids. Phosphodiesters (PDEs), on the other hand, are the products of phospholipid breakdown and both PMEs and PDEs serve as markers for phospholipid membrane turnover [11], [14]. Furthermore, the chemical shift separation of Pi and β-ATP relative to PCr gives estimates of intracellular pH and magnesium (Mg${}^{2+}$) concentration [15]. Despite the wealth of information contained in the ^31^P-spectrum, the clinical application of ^31^P-MRS is still in its infancy. Due to the low MR sensitivity of ^31^P the method remains experimental and so far few clinical studies have used this technique [7]. Advances in ^31^P-MRS are primarily made in the preclinical field where ultra-high magnetic field strengths (≥ 7T) provide improved signal-to-noise ratio (SNR) and shortened longitudinal relaxation time ($T_{1}$) of ^31^P metabolites which in combination provide an SNR per unit time sufficiently for in vivo application [16]. Collectively, these efforts aim to demonstrate the utility of the method and improve the technique sufficiently to make it clinically feasible [17].

Typically, dual-tuned ${}^{1}$H/^31^P RF coils are used to allow the use of the stronger proton signal for fast image-based localization and main field (B${}_{0}$) shimming. Shimming based on the ^31^P signal would be problematic given its low SNR. Furthermore, dual-tuned ${}^{1}$H/^31^P RF coils enable fast anatomical (proton-based) image acquisition for VOI placement. This setup improves SNR for ^31^P-MRS through accurate VOI placement and high quality localized shimming [18]. VOI placement is important to reduce partial volume effects and to limit investigations to regions relevant to the biological question being studied.

Preclinical animal research aids clinical research by imitating phenotypes of various pathological human conditions, with mice accounting for the majority of animals used [19]. Most mouse MRI/S studies are performed under general anaesthesia [20], [21], [22] despite the fact that anaesthesia is known to affect brain function [23], [24], microstructure [25], metabolism [26], and metabolites detectable by MRS [27]. Nevertheless, anaesthesia protocols are given and recommended by MRS experts [20] based on the argument that the reduction of stress and motion artefacts, using anaesthesia, minimizes data corruption. Furthermore, the logistical challenges related to awake scans are considerable [28]. However, human MRI or MRS studies rarely subject participants to general anaesthetics. The common use of general anaesthesia in preclinical research, therefore, limits the degree of comparability of preclinical MRS to clinical research findings [29].

Recently, awake mouse habituation protocols [30], [31], [32] have been developed showing that both male and female mice can successfully be trained for extended awake MRI sessions. The protocol consists of surgically implanting a head collar (various designs exist e.g. [33], [34]) to physically restrain the mice followed by repeated habituation sessions to familiarize the animal with the head collar restraint and acoustic noise of the scanner. This protocol avoids high stress levels and enables high quality MR data acquisition free of motion artefacts without the physiological pertubations from anaesthesia.

**Fig. 1:**
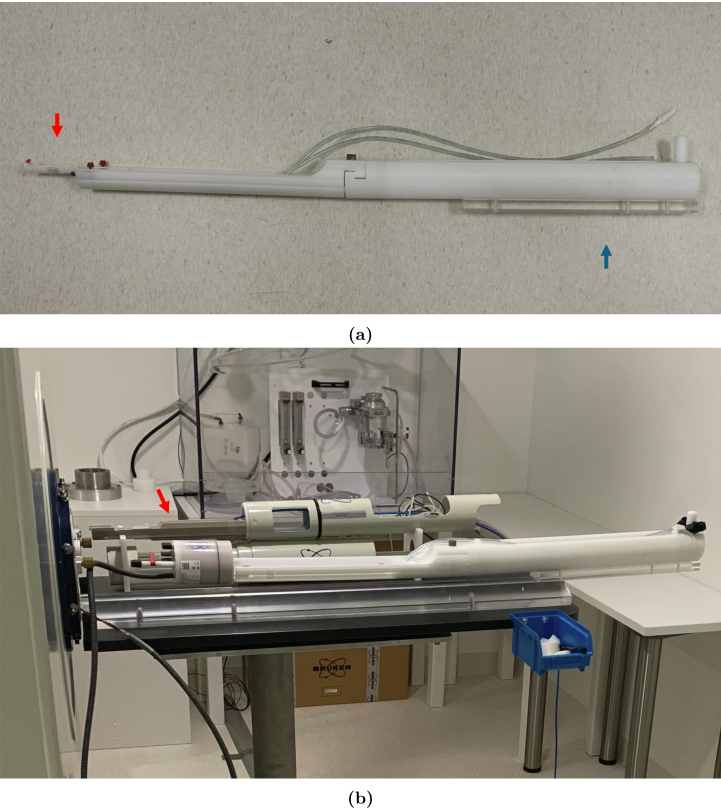
(a) Bruker’s rig system is depicted here. The rear section (blue arrow) features a short segment used to attach to the rail, extending 30 cm into the scanner. The front part (red arrow), where the coil is positioned, is located at the end of the rig system. (b) Bruker’s rig setup is placed on the rail, which is positioned on the table in front of the scanner bore and extends 30 cm into the scanner. The volume coil is shown here to demonstrate that it is positioned at the front part of this setup (red arrow), suspended similarly to a diving board. Bruker’s rig-rail setup for mouse ${}^{1}$H/^31^P-MRS.

With Bruker’s dual-tuned ${}^{1}$H/^31^P RF volume coil (T20025V3) follows a rig-rail setup (see Fig. 1). The rail is positioned on the table in front of the scanner and continues 30 cm into the scanner in contiguity with the floor of the scanner bore. The rig’s rear part runs on the rail, while the coil and the mice are placed on the front part of the rig, suspended similarly to a “diving board” or cantilever jump stand (see Fig. 1(b)). The rig is positioned into the scanner by sliding it on the rail. With the table being attached to the front end of scanner and the rail mounted to the table this setup creates a chain where scanner vibrations are easily transferred and even amplified up to the tip of the cantilever that holds the RF-coil. The root cause of these vibrations are primarily strong gradient pulses or mechanical resonances from the surroundings (e.g. from the pump action of the magnet cold head).

When designing a coil suspension setup suitable for awake animal MRI/S and the customized animal cradle, special care must be taken to eliminate such coil vibrations during scans as these effects are detrimental to data quality. Currently, no commercially available setups provide all of these desired functionalities and the flexibility for users to modify according to specific experimental requirements.

The scope of this article is, therefore, to present a Mouse Cradle Suspension System (MCSS) for MRS, circumventing these limitations and thereby enabling both awake and anaesthetized ${}^{1}$H/^31^P-MRS scans. The design features (1) a suspension system that avoids amplification of the scanner vibrations, (2) a rail system compatible with Bruker’s dual-tuned ${}^{1}$H/^31^P RF volume coil (T20025V3) and the RAPID dual-tuned ${}^{1}$H/^31^P surface coil (O-XL-HL-094-01843) and (3) a new mouse cradle compatible with a head collar modified from the design by Han et al. [33] previously used for awake mice habituation [32]. The described setup allows both awake and anaesthetized scans.

## 2 Hardware description

**Fig. 2:**
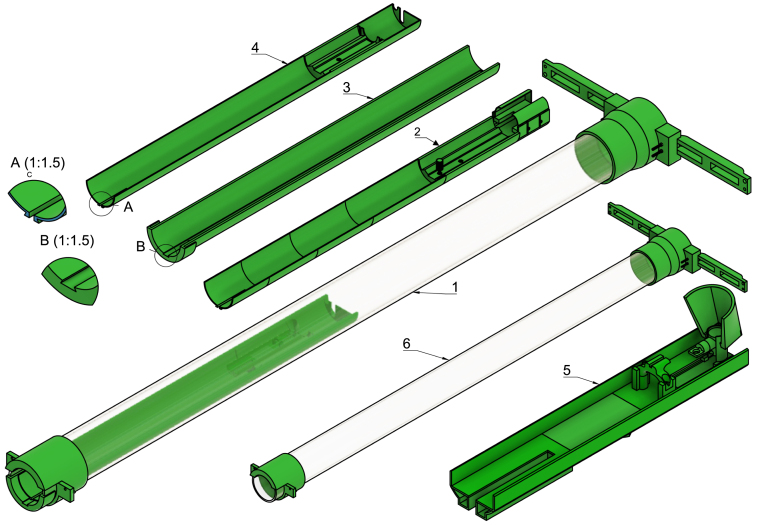
3D rendered view of the Mouse Cradle Suspension System (MCSS). 1: Fully assembled MCSS, 2: Inner rail (surface coil) 3: Outer rail, 4: Inner rail (volume coil), 5: Mouse cradle and 6: Suspension system. The components illustrated here are not to scale.

The MCSS design focuses on stability and simplicity of hardware, assembly, adjustability, and low cost. The resulting setup comprises three major components. The first is a suspension system consisting of an acrylic tube mounted to the front and rear attachment sites used for Bruker Biospin’s rodent brain cryo-array coil system (see Fig. 2, item 6). The suspended tube ensures precise and stable positioning of the RF coil in the scanner isocenter. The second component comprises an inner and outer rail, with the inner rail sliding onto the outer rail. There are two versions of the inner rail: one for the volume coil and one for the surface coil (see Fig. 2, item 2–4). The inner rails have a bed (for the volume coil) or a socket (for the surface coil), with a mouse cradle rail in front allowing flexible positioning of a mouse cradle (see supplementary fig. 23). The third component comprises a new mouse cradle with the following integrated into its design; (1) a head-stage designed to hold a head collar design modified from the one by Han et al. [33] compatible with both anaesthetized and awake scan setups, (2) an easily attached and detached whisker stimulation tube for functional MRI (fMRI) experiments (see supplementary fig. 25), (3) an adjustable anaesthesia tube and (4) a water container with water flow integrated into the cradle plate for optimal body temperature regulation during anaesthesia.

With the coil and mouse cradle placed on the inner rail, the entire inner and outer rail go flush into the acrylic tube of the suspension system. The Bruker Biospec 94/20 scanner’s isocenter is 74.8 cm from the bore front, and the position of the inner rail on the outer rail can be adjusted, allowing the coil and mouse cradle to be easily positioned into the scanner’s isocenter. All of this presents an easy-to-use and adjustable setup for MRS experiments on any organs of interest in mice in both awake and anaesthetized states (see Fig. 2, item 1).

This setup is therefore useful for the following experiments:

• Awake mice ${}^{1}$H/^31^P MRS of brain and other organs.
• Anaesthetized mice ${}^{1}$H/^31^P MRS of brain and other organs.
• Awake mouse whisker stimulation ${}^{1}$H/^31^P magnetic resonance spectroscopy and ${}^{1}$H fMRI.

## Design files

All components are designed using autodesk fusion 360 (Autodesk, USA). All files for 3D-printing are available as CAD and stl-files (see Table 3.1). Due to the limited print height of 3D printers, some components of the outer rail, the inner rail (surface coil), and the inner rail (volume coil) are made in multiple parts.

## 3 Design files summary

**Table 3.1:** Design files.

Design filename	File type	Open source license	File location	Major component
Front acrylic tube mount	CAD	CC BY 4.0	[35]	Suspension system
Rear acrylic tube mount	CAD	CC BY 4.0	[35]	Suspension system
Wings	CAD	CC BY 4.0	[35]	Suspension system
Rail holder	CAD	CC BY 4.0	[35]	Suspension system
Front acrylic tube mount	stl	CC BY 4.0	[35]	Suspension system
Rear acrylic tube mount	stl	CC BY 4.0	[35]	Suspension system
Wings	stl	CC BY 4.0	[35]	Suspension system
Rail holder	stl	CC BY 4.0	[35]	Suspension system
Outer rail 200 mm	CAD	CC BY 4.0	[35]	Outer rail
Outer rail 152 mm	CAD	CC BY 4.0	[35]	Outer rail
Outer rail socket	CAD	CC BY 4.0	[35]	Outer rail
Outer rail (thread)	CAD	CC BY 4.0	[35]	Outer rail
Outer rail 200 mm	stl	CC BY 4.0	[35]	Outer rail
Outer rail 152 mm	stl	CC BY 4.0	[35]	Outer rail
Outer rail socket	stl	CC BY 4.0	[35]	Outer rail
Outer rail (thread)	stl	CC BY 4.0	[35]	Outer rail
Inner rail 200 mm	CAD	CC BY 4.0	[35]	Common parts for the inner rails
Inner rail 152 mm	CAD	CC BY 4.0	[35]	Common parts for the inner rails
Inner rail (front)	CAD	CC BY 4.0	[35]	Common parts for the inner rails
Screw head	CAD	CC BY 4.0	[35]	Common parts for the inner rails
Inner rail 200 mm	stl	CC BY 4.0	[35]	Common parts for the inner rails
Inner rail 152 mm	stl	CC BY 4.0	[35]	Common parts for the inner rails
Inner rail (front)	stl	CC BY 4.0	[35]	Common parts for the inner rails
Screw head	stl	CC BY 4.0	[35]	Common parts for the inner rails
Backplate (coil bed)	CAD	CC BY 4.0	[35]	Inner rail volume coil
Coil bed	CAD	CC BY 4.0	[35]	Inner rail volume coil
Mouse cradle rail	CAD	CC BY 4.0	[35]	Inner rail volume coil
Backplate (coil bed)	stl	CC BY 4.0	[35]	Inner rail volume coil
Coil bed	stl	CC BY 4.0	[35]	Inner rail volume coil
Mouse cradle rail	stl	CC BY 4.0	[35]	Inner rail volume coil
Surface coil socket	CAD	CC BY 4.0	[35]	Inner rail surface coil
Surface coil rail	CAD	CC BY 4.0	[35]	Inner rail surface coil
Mouse cradle rail (extended)	CAD	CC BY 4.0	[35]	Inner rail surface coil
Inner rail 133.7 mm	CAD	CC BY 4.0	[35]	Inner rail surface coil
Surface coil socket	stl	CC BY 4.0	[35]	Inner rail surface coil
Surface coil rail	stl	CC BY 4.0	[35]	Inner rail surface coil
Mouse cradle rail (extended)	stl	CC BY 4.0	[35]	Inner rail surface coil
Inner rail 133.2 mm	stl	CC BY 4.0	[35]	Inner rail surface coil
Head collar	CAD	CC BY 4.0	[35]	Mouse cradle
Mouse cradle (top plate)	CAD	CC BY 4.0	[35]	Mouse cradle
Mouse cradle (rear bottom)	CAD	CC BY 4.0	[35]	Mouse cradle
Water container	CAD	CC BY 4.0	[35]	Mouse cradle
Anaesthesia outer tube	CAD	CC BY 4.0	[35]	Mouse cradle
Anaesthesia inner tube	CAD	CC BY 4.0	[35]	Mouse cradle
Anaesthesia mask	CAD	CC BY 4.0	[35]	Mouse cradle
Anaesthesia tip	CAD	CC BY 4.0	[35]	Mouse cradle
Whisker tube	CAD	CC BY 4.0	[35]	Mouse cradle
Head collar	stl	CC BY 4.0	[35]	Mouse cradle
Mouse cradle (top plate)	stl	CC BY 4.0	[35]	Mouse cradle
Mouse cradle (rear bottom)	stl	CC BY 4.0	[35]	Mouse cradle
Water container	stl	CC BY 4.0	[35]	Mouse cradle
Anaesthesia outer tube	stl	CC BY 4.0	[35]	Mouse cradle
Anaesthesia inner tube	stl	CC BY 4.0	[35]	Mouse cradle
Anaesthesia mask	stl	CC BY 4.0	[35]	Mouse cradle
Anaesthesia tip	stl	CC BY 4.0	[35]	Mouse cradle
Whisker tube	stl	CC BY 4.0	[35]	Mouse cradle

### Rail holder

Two rail holders can be placed into the table rail, which are used to hold the entire rail system (see Fig. 19 step 9).

### Front acrylic tube mount

**Fig. 3:**
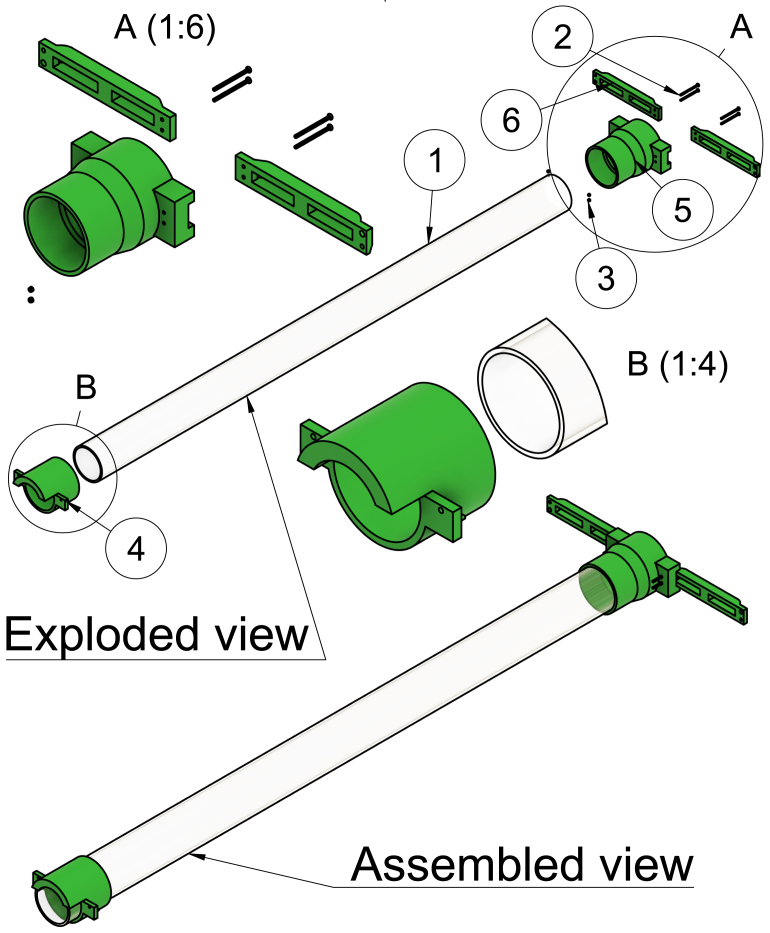
3D rendered exploded and assembled view of the suspension system. 1: Acrylic tube, 2: M3 40 mm stainless steel bolt, 3: M3 locknut in stainless steel, 4: Front acrylic tube mount, 5: Rear acrylic tube mount and 6: Wings.

This part has a socket for the acrylic tube to be inserted into. This avoids the need for drilling into the acrylic tube which is so brittle that cracks easily form (see Fig. 3 item 4).

### Rear acrylic tube mount

The acrylic tube is inserted into this part from the rear end of the scanner. It has two attachment sites for the wings, which ensure mechanical stability. The attachment sites have two small holes for connecting the wings with bolts and locknuts (see Fig. 3 item 5).

### Wings

Besides being connected to the rear acrylic tube mount, the other end of this 3D-printed part has a small and a large hole. The large hole is for the peg of Bruker Biospin’s rear attachment site to be inserted into, and the small hole is for the brass bolt to secure the wings tightly to the rear attachment site (see Fig. 3 item 6).

### Inner rail 200 mm and 152 mm

Both parts are similar in function and together ensure the needed length for the inner rail (volume coil) and inner rail (surface coil) (see Fig. 5 item 5–6, and Fig. 6 item 4–5).

### Inner rail (front)

This is the front part for the inner rail (volume coil) and inner rail (surface coil). It has a small gap that allows the inner rail to slide, while an M5 nylon bolt is loosely screwed to the outer rail just below it. Once the inner rail is positioned correctly, the inner rail is fixed to the outer rail by tightening the bolt (see Fig. 5 item 7, and Fig. 6 item 7).

### Screw head

**Fig. 4:**
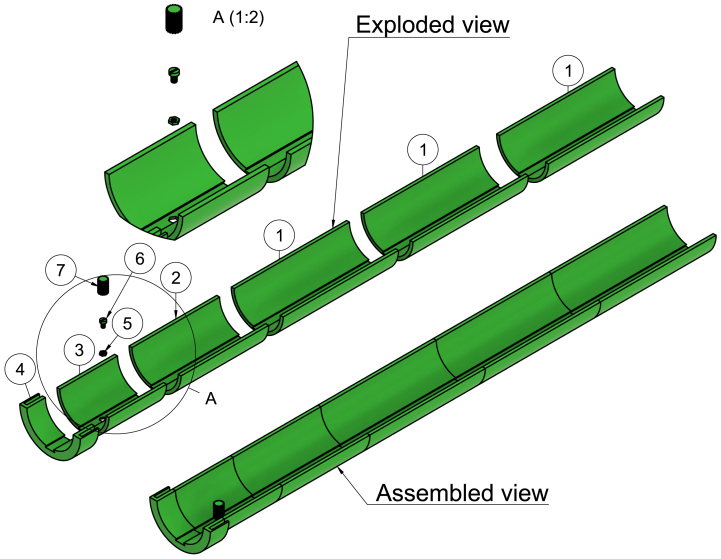
3D rendered exploded and assembled view of the outer rail. 1: Outer rail 200 mm, 2: Outer rail 152 mm, 3: Outer rail (thread), 4: Outer rail socket, 5: M5 nylon hexagon shaped nut, 6: M5 nylon bolt, 7: Screw head.

**Fig. 5:**
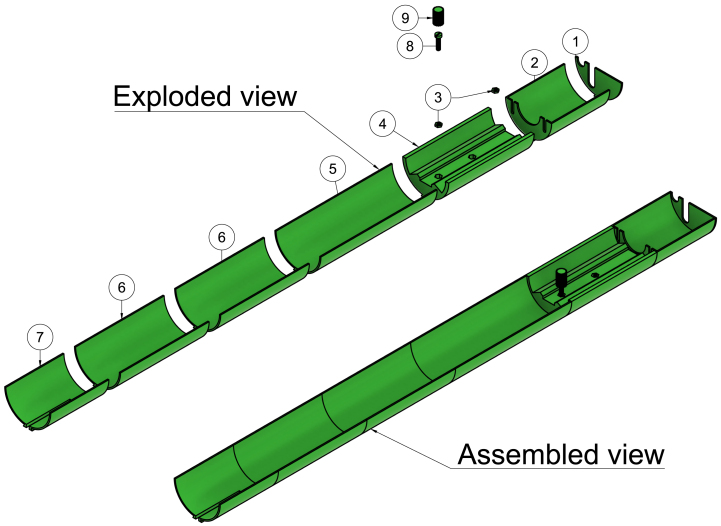
Exploded and assembled 3D rendered view of the inner rail (volume coil). 1: Backplate (coil bed), 2: coil bed, 3: M5 nylon nut, 4: Mouse cradle rail, 5: Inner rail 200 mm, 6: Inner rail 152 mm, 7: Inner rail (front), 8: M5 nylon bolt with cylinder head, 9: Screw head.

**Fig. 6:**
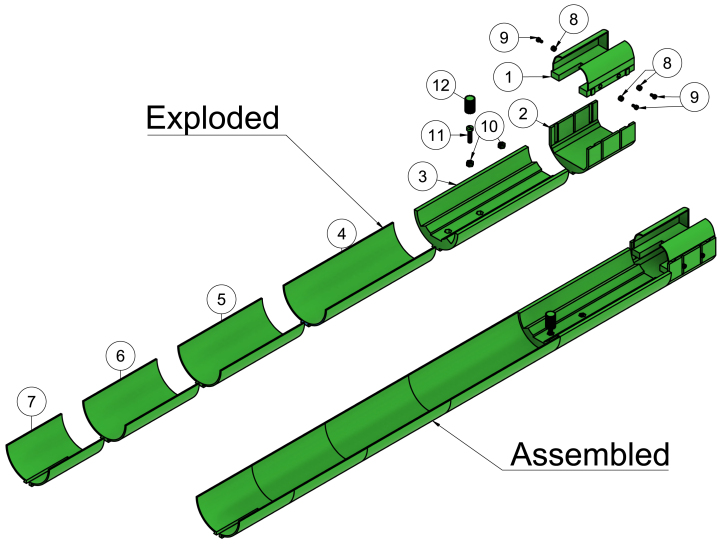
Exploded and assembled 3D rendered view of the inner rail for the dual tuned surface coil and the components required. 1: Surface coil socket, 2: Surface coil rail, 3: Mouse cradle rail (extended), 4: Inner rail 200 mm, 5: Inner rail 152 mm, 6: Inner rail 133.7 mm, 7: Inner rail (front), 8: M3 hexagon nylon nut, 9: M3 nylon bolt, 10: M5 hexagon nylon nut, 11: M5 nylon bolt, 12: Screw head.

The screw head is squeezed onto a M5 nylon bolt and gives a cheap, easy and ergonomic solution for the screw function. This screw is used to fix the inner rails to the outer rail (see Fig. 4 item 7) and to fix the mouse cradle to the inner rails (see Fig. 5 item 9, and Fig. 6 item 12).

### Outer rail 200 mm and 152 mm

These parts are similar in function and altogether give the outer rail its needed length (see Fig. 4 item 1 and 2).

### Outer rail (thread)

This part of the rail is located at the front end of the outer rail. It has a hexagon-shaped hole for a nylon nut to be inserted and glued firmly. This is where the inner rail (both versions) will be screwed down to prevent it from moving once it has been positioned correctly (see Fig. 4 item 3).

### Outer rail socket

The outer rail needs to be fixed to the acrylic tube to avoid any sort of movement while scanning. The outer rail socket is the front part of the outer rail and has a socket that tightly clamps to the acrylic tube (see Fig. 4 item 4).

### Coil bed and backplate (coil bed)

These two 3D-printed parts ensure a snug fit for the ${}^{1}$H/^31^P volume coil to be inserted while allowing access for the mouse cradle to slide into the coil (see Fig. 5 item 1 and 2).

### Mouse cradle rail

This section of the inner rail has two hexagon shaped holes for M5 nylon nuts to be glued into (see Fig. 5 item 4). The mouse cradle is fixed to the nylon nut furthest from the volume coil while preparing and positioning the mouse on the mouse cradle. Once the mouse has been prepared, the mouse cradle slides into the volume coil and is fixed to the mouse cradle rail using the nylon nut closest to the volume coil. Its rail system continues flush with the volume coil’s inner surface and ensures a smooth sliding mechanism for the mouse cradle.

### Inner rail 132.7 mm

This part is a shorter version of the inner rail 200 mm and the inner rail 152 mm, and serve the same purpose (see Fig. 6, items 4-6).

### Mouse cradle rail (extended)

This 3D-printed part is similar to the mouse cradle used for the inner rail (volume coil). However, due to the surface coil being longer than the volume coil, the mouse cradle has been extended by 20 mm (see Fig. 6 item 3).

### Surface coil socket

This 3D-printed part has a socket for the dual tuned ${}^{1}$H/^31^P surface coil and ensures a snug fit. The sides have stubs for the vertical sliding mechanism when placed into the surface coil rail. There are three hexagon shaped holes on the sides for M3 nylon nuts to be glued into (see Fig. 6 item 1 and 8).

### Surface coil rail

The surface coil rail is next to the mouse cradle rail and has vertical rails that allow the surface coil socket to slide up and down. The square notch ensures room for the mouse cradle when moving the mouse head beneath the surface coil centre. Once the mouse head is positioned at the coil’s centre, the surface coil socket is lowered further down toward the mouse head and locked in position using M3 nylon bolts.(see Fig. 6 item 2 and 9).

### Head collar

**Fig. 7:**
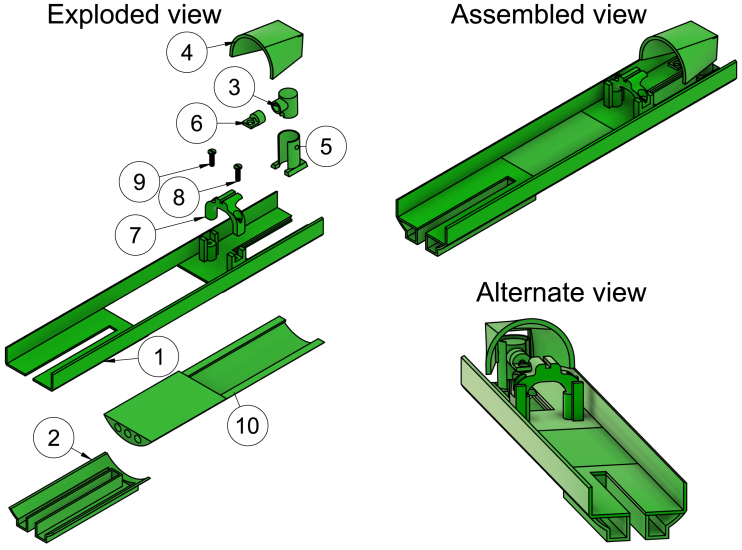
3D rendered exploded and assembled view of the mouse cradle. 1: Mouse cradle (top plate), 2: Mouse cradle (rear bottom), 3: Anaesthesia inner tube, 4: Anaesthesia mask, 5: Anaesthesia outer tube, 6: Anaesthesia tip, 7: Head collar. 8-9: M2 nylon bolt, 10: Water container.

This head collar is a modified version of the design from Han et al. [33] (see Fig. 7 item 7). The previous version had threads below the tips of the collar, and the mouse collar was fixed to a bed by a bolt going through the bed plate and into the threads on the head collar. This meant that if the thread wore down, the mouse could no longer be fixed and thus used for awake scans. This version has holes on both sides of the collar, allowing an M2 nylon bolt to pass through and be fixed to the mouse cradle’s head stage, where the threads are integrated. This means if the threads on the mouse cradle wear down, only a new bed needs to be printed for the repair.

### Mouse cradle (top plate)

This 3D-printed part has a rail at the front end where the head stage is located. This enables the anaesthesia outer tube to adjust its position according to the mouse head fixed to the head stage (see Fig. 7 item 1).

### Water container

This is the only part made of acrylonitrile styrene acrylate (ASA) filament. This type of filament is more heat resilient. It is positioned in the large gap in the middle of the mouse cradle (top plate). It has two holes for water in and outflow and a hole going all the way through, giving passage for the anaesthesia tube (see Fig. 7 item 10).

### Mouse cradle (rear bottom)

This 3D-printed part is glued next to the water container on the rear side of the mouse cradle (top plate). Its cavities further guide the water and anaesthesia tubes from the water container to the rear end of the mouse cradle (see Fig. 7 item 2).

### Anaesthesia outer and inner tube

The setup for anaesthesia has an outer and inner tube, with the outer tube sliding back and forth on the mouse cradle (top plate), and the inner tube sliding vertically up and down along the outer rail’s inner wall. The inner and outer tubes have a snug fit to ensure that the inner tube can stay at any position it is at. Likewise, for the exit of anaesthesia gas, a perpendicular tube extends outward toward the mouse from the top part of the inner tube (see Fig. 7 item 3 and 5).

### Anaesthesia tip

The anaesthesia tip is glued to the inner rail’s short tube that points toward the mouse. It has a large hole for the mouse’s upper incisors to be positioned in, with a very small hole in front of it where the anaesthesia gas exits (see Fig. 7 item 6).

### Anaesthesia mask

A 3D-printed mask is used to contain the anaesthesia gasses around the mouse head. This mask has two small sockets for the small pegs on the anaesthesia outer tube to be inserted into and give the mask an easy-to-use on-and-off hinge joint (see Fig. 7 item 3).

### Whisker tube

The whisker tube is clamped in between the mouse cradle’s side wall and the head stage. The tube has a hole for the air tube and fans out to ensure an evenly distributed puff of air to the mouse’s whiskers (see supplementary fig. 25).

## Bill of materials

All non-3D-printed parts are commercial off-the-shelf components that can be purchased in most hardware stores. The cost of each 3D-printed element is derived according to their weight and the price for 1 kg of polylactic acid (PLA) or ASA filament (22.85 and 24.2 euros, respectively) (see Table 3.2).

**Table 3.2:** Bill material for the MCSS.

Designator	Component	Number	Cost per unit (euros)	Total cost (euros)	Source of materials	Material type
200019	Mount acrylic tube (front)	1	4,12	4,12	[36]	PLA
200019	Mount acrylic tube (rear)	1	0,34	0,34	[36]	PLA
200019	Wings for rear acrylic tube mount	2	0,26	0,52	[36]	PLA
200019	Outer rail socket	1	0,02	0,02	[36]	PLA
200019	Outer rail 200	3	2,17	6,51	[36]	PLA
200019	Outer rail 152	1	1,65	1,65	[36]	PLA
200019	Screw head	2	0,07	0,13	[36]	PLA
200019	Outer rail nuts	1	1,10	1,10	[36]	PLA
200019	Back plate	1	0,11	0,11	[36]	PLA
200019	Coil bed	1	0,84	0,84	[36]	PLA
200019	Inner rail front	1	0,73	0,73	[36]	PLA
200019	Inner rail 133.7	1	0,95	0,95	[36]	PLA
200019	Inner rail 152	1	1,15	1,15	[36]	PLA
200019	Inner rail 200	1	1,51	1,51	[36]	PLA
200019	Mouse cradle (top plate)	1	2,15	2,15	[36]	PLA
200019	Mouse cradle (rear bottom)	1	0,26	0,26	[36]	PLA
200019	Mouse cradle (top plate)	1	0,34	0,34	[36]	PLA
200019	Anaesthesia inner tube	1	0,02	0,02	[36]	PLA
200019	Anaesthesia outer tube	1	0,01	0,01	[36]	PLA
200019	Whisker tube	1	0,01	0,01	[36]	PLA
200019	Anaesthesia mask	1	0,05	0,05	[36]	PLA
200019	Screw head	1	0,05	0,05	[36]	PLA
200602	Water container	1	0,16	0,16	[37]	ASA
436572	Super glue power gel Loctite	4	4,81	19,24	[38]	Resin adhesive
CL645123	2-K instant adhesive Loctite 3090	1	32,50	32,50	[39]	Resin adhesive
525–723	M5 nut	3	0,30	0,90	[40]	Nylon
527–763	M5 screw cylinder head 20 mm	2	0,60	1,20	[41]	Nylon
525–723	M3 nut	6	0,24	1,44	[42]	Nylon
527–763	M3 screw cylinder head 7 mm	3	0,24	0,72	[43]	Nylon
RTPMPX10000502	Acrylic tube	1	124,00	124,00	[44]	Acrylic
560–631	M3 40 mm screw	4	0,02	0,09	[43]	Stainless steel
524–281	M3 locknut	4	0,00	0,02	[45]	Stainless steel
	Grand total			201,17		

## 4 Build instructions

### Settings for 3D-printing

All 3D-printed parts were printed on a Prusa i3 MK3S+ 3D printer capable of printing up to a height of 210 mm. The design of the outer and inner rail is therefore comprised of several 3D-printed parts that are glued together, and will be described in further detail in this section. Stl-files are exported to Prusaslicer 2.4.2 (Version 2.8.0+MacOS-arm64) where the setup for 3D printing was made. Slice resolution was 0.2 mm (quality), with the first layer 0.2 mm in the layer height. The infill was set at 15%. The 3D-printed elements were made of polylactic acid (PLA) plastic filaments with a diameter of 1.75 mm. PLA is suitable for this project because it does not emit toxic gasses and has minimal warping issues. The extruder nozzle temperature was set to 205 °C for PLA, and the platform to 60 °C. The water container was made of Acrylonitrile styrene acrylate (ASA) due to its higher heat resilience at 105 °C compared to PLA at 40 °C. The temperature for ASA was 260 °C for the nozzle and 110 °C for the platform. To ensure a watertight 3D print, the layer height was set at 0.1 mm with the extrusion multiplier set to 1.02 which ensures no gaps between each print layer. With these settings, a gcode-file was generated and imported into the Prusa i3 MK3S+ 3D printer (0.4 mm nozzle) ready for print. Before printing, the platform was wiped off with 70% ethanol to de-grease the surface to ensure that the first print layer easily sticks to the platform.

## Suspension system

**Fig. 8:**
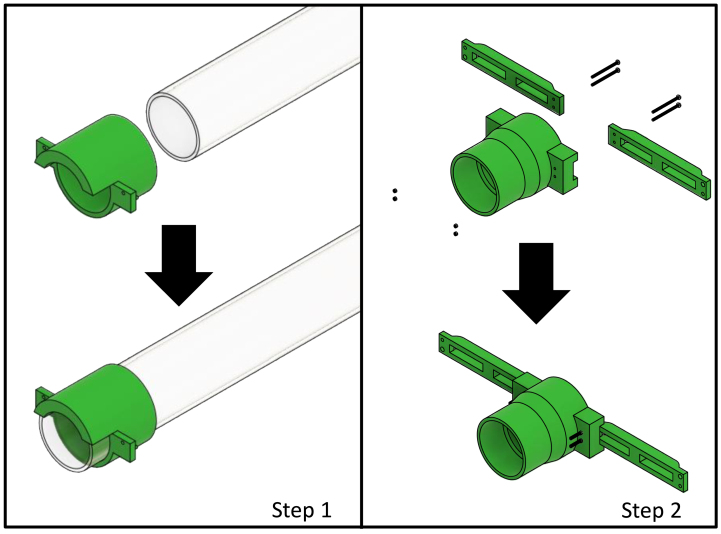
Build instruction steps for the suspension system.

The front acrylic tube mount has the acrylic tube squeezed into its socket (see Fig. 8 step 1). Two wings are tightly screwed to the rear acrylic tube mount with M3 bolts and lock nuts (see Fig. 8 step 2).

## Outer rail

**Fig. 9:**
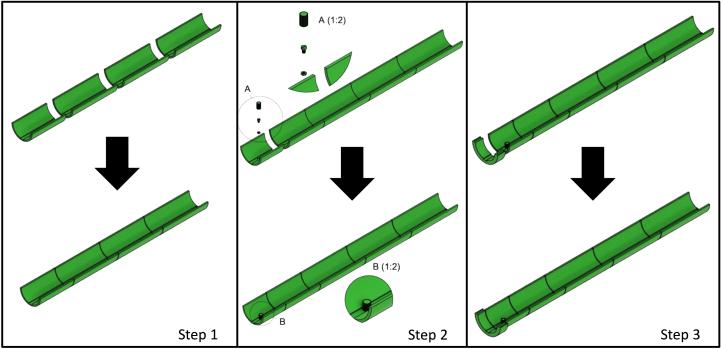
Build instruction steps for the outer rail.

Glue together the following parts from the rear, starting with the three outer rails 200 mm and one outer rail 152 mm (see Fig. 9 step 1). Glue a single M5 nylon nut down to the hexagon-shaped hole in the outer rail (thread). Then glue the outer rail (thread) to the outer rail 152 mm. Squeeze the screw head down to the M5 nylon bolt with a cylinder-shaped head (see Fig. 9 step 2). Finally, glue the outer rail socket to the outer rail (thread) (see Fig. 9 step 3). Finish off by sanding down the inner side of the outer rail where the inner rail will slide along. Make sure to even out any parts of the M5 nylon nut that may not be flush with the outer rail.

## Inner rail (volume coil)

**Fig. 10:**
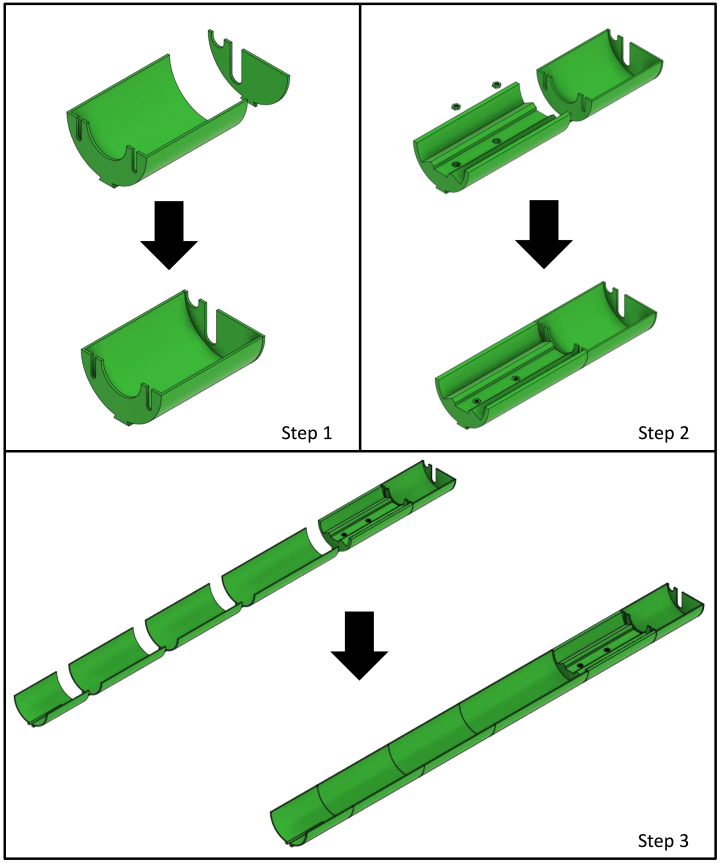
Build instruction steps for the inner rail (volume coil).

Apply glue to the coil bed on the surface where the backplate (coil bed) will be attached (see Fig. 10 step 1). Screw two M5 nylon nuts onto a bolt. Make sure the two nuts are close and tight next to each other and at the tip of the bolt. This ensures the nut threads are aligned with each other. Then apply glue on their sides and, with the nuts at the tip of the bolt, insert into the hexagon-shaped hole in the mouse cradle rail and let it dry (for visual aid, see supplementary fig. 24). Then, glue the mouse cradle rail to the front side of the coil bed (see Fig. 10 step 2). Following the mouse cradle rail, glue two inner rails 200 mm to it, followed by one inner rail 152 mm, and finally one inner rail (front) (see Fig. 10 step 3). The glue used for the inner rail assembly is Loctite super glue power gel. Note: When gluing the parts together flip the 3D-printed parts upside down facing a flush table top. This makes it easier for the parts to be glued flush together. Use sandpaper on the bottom side where the inner rail will slide along the outer rail to give it a smooth finish.

## Inner rail (surface coil)

**Fig. 11:**
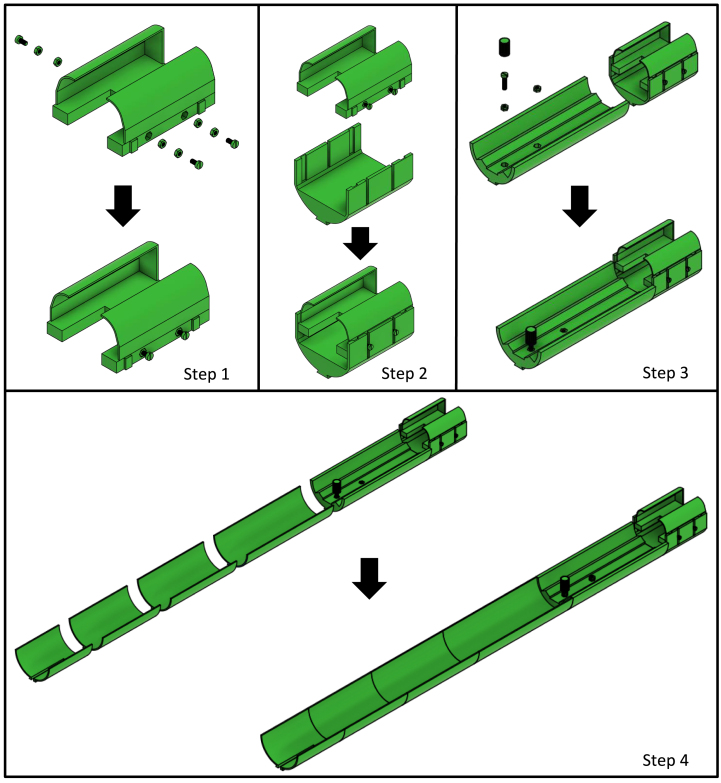
Build instruction steps for the inner rail (surface coil).

Similar to step 2 in Section 4 (Inner rail (volume coil)), but with M3 nylon nuts, insert the M3 nylon nuts into the three hexagon-shaped holes positioned on the sides of the surface coil socket (see Fig. 11 step 1). Slide the surface coil socket into the surface coil rail (see Fig. 11 step 2). Glue the mouse cradle rail (extended) to the front side of the surface coil rail. Similarly, the M5 nylon nuts are glued to the mouse cradle rail (extended) exactly the same way as done with the mouse cradle rail (see Section 4, (Inner rail (volume coil)) step 2). Glue inner rail 200 mm, inner rail 152 mm and inner rail 133.7 mm in continuation of the mouse cradle rail (extended) (see Fig. 11 step 4).

## Mouse cradle

**Fig. 12:**
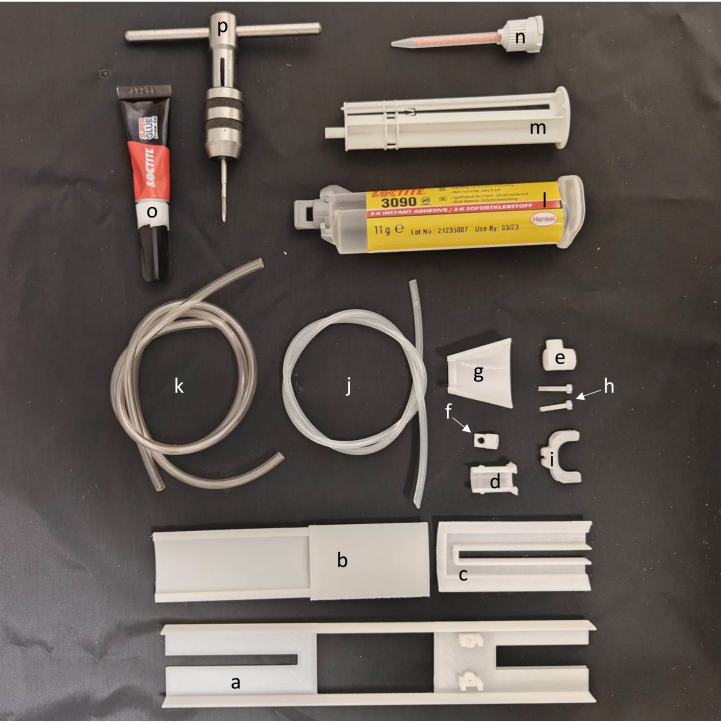
Parts for the mouse cradle, a: Mouse cradle (top plate), b: Water container, c: Mouse cradle (rear bottom), d: Anaesthesia outer tube, e: Anaesthesia inner tube, f: Anaesthesia tip, g: Anaesthesia mask, h: M2 nylon bolt, i: Head collar, j: 3.1 mm diameter anaesthesia tube (25 cm long), k: 2 x 4 mm diameter water tube (20 cm long), l-n: 2-K instant adhesive Loctite 3090, o: Loctite super glue power gel and p: M2 thread cutter.

**Fig. 13:**
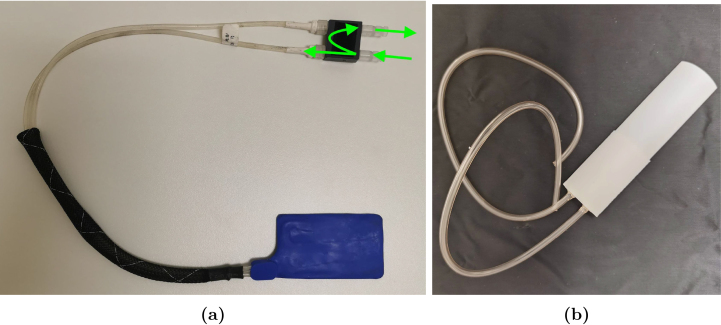
(a) Bruker’s water flow system for thermoregulation with a green arrow illustrating the water flow shunting away from the blue heating pad. (b) The waterflow system integrated in the mouse cradle.

**Fig. 14:**
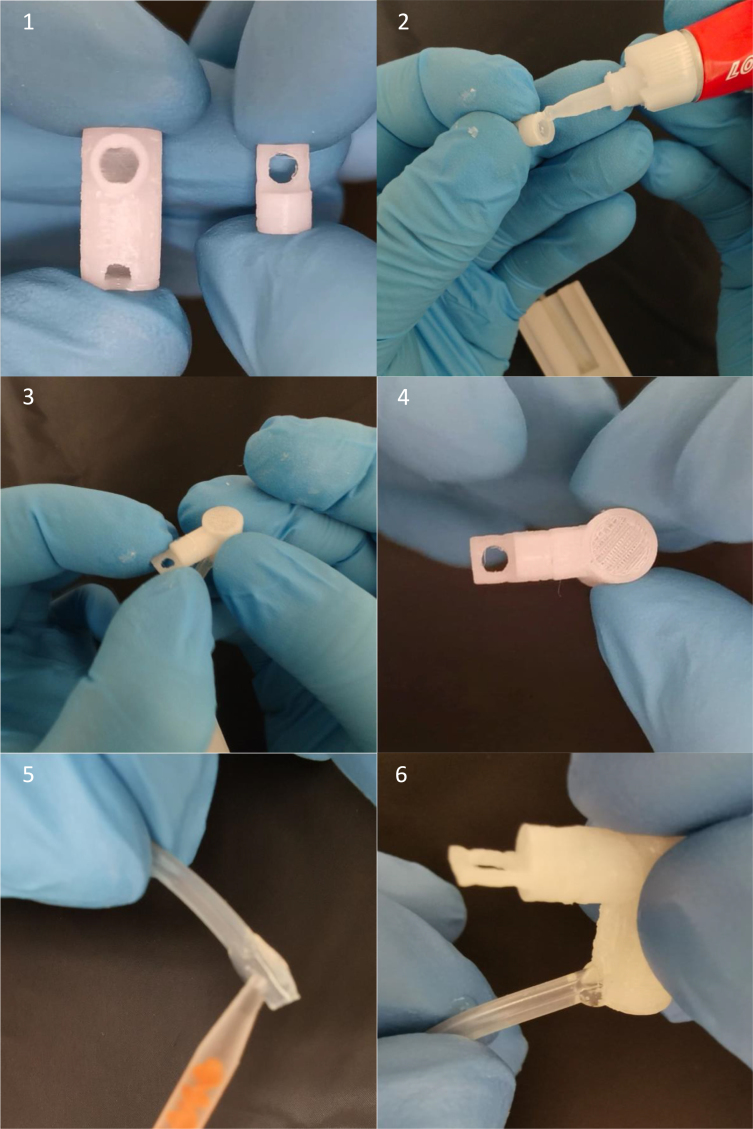
Step 1–4: Anaesthesia inner tube and anaesthesia tip are glued together. Step 5–6: 3.1 mm diameter tube (25 cm long) has 2-K instant adhesive Loctite 3090 glued to its sides and inserted into the bottom hole in the anaesthesia inner tube. Do not wipe off any over oozing glue, since that will diminish the air tightness. Compared to regular Loctite gel glue, the 2-K instant adhesive Loctite 3090 glue is better at filling gaps and creating a watertight/airtight seal, hence its use for anaesthesia and water tubes.

**Fig. 15:**
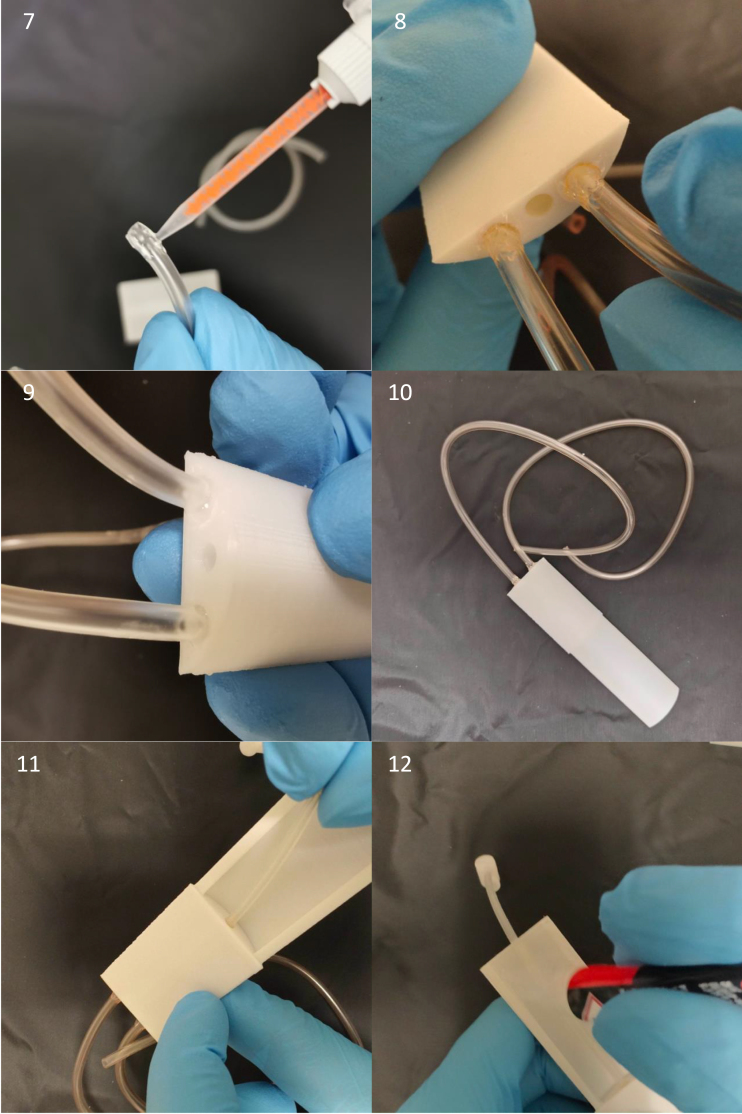
Step 7–10: Apply 2-K instant adhesive Loctite 3090 glue to both water tubes (20 cm long, 4 mm diameter) and insert into the water container and let dry for 5 min. Step 11: Guide the anaesthesia tube all the way through the middle hole of the water container. Step 12: Apply glue on the sides of the water container.

**Fig. 16:**
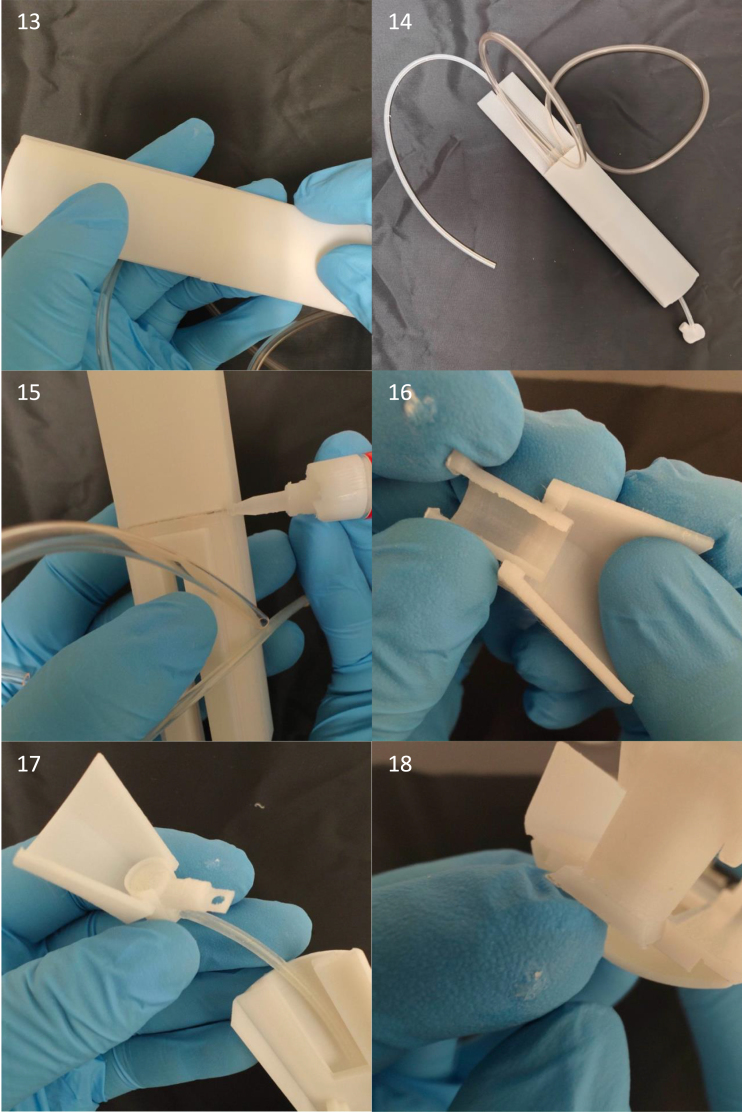
Step 13–14: Squeeze the water container to the mouse cradle (top plate) and hold for a couple of minutes and let it dry for 5 min. Step 15: Glue the mouse cradle (rear bottom) to the mouse cradle (top plate). Step 16–17: Click the anaesthesia mask into the anaesthesia outer tube. Next slide the anaesthesia inner tube into the anaesthesia outer tube. Step 18: Slide the anaesthesia outer into the mouse cradle (top plate).

**Fig. 17:**
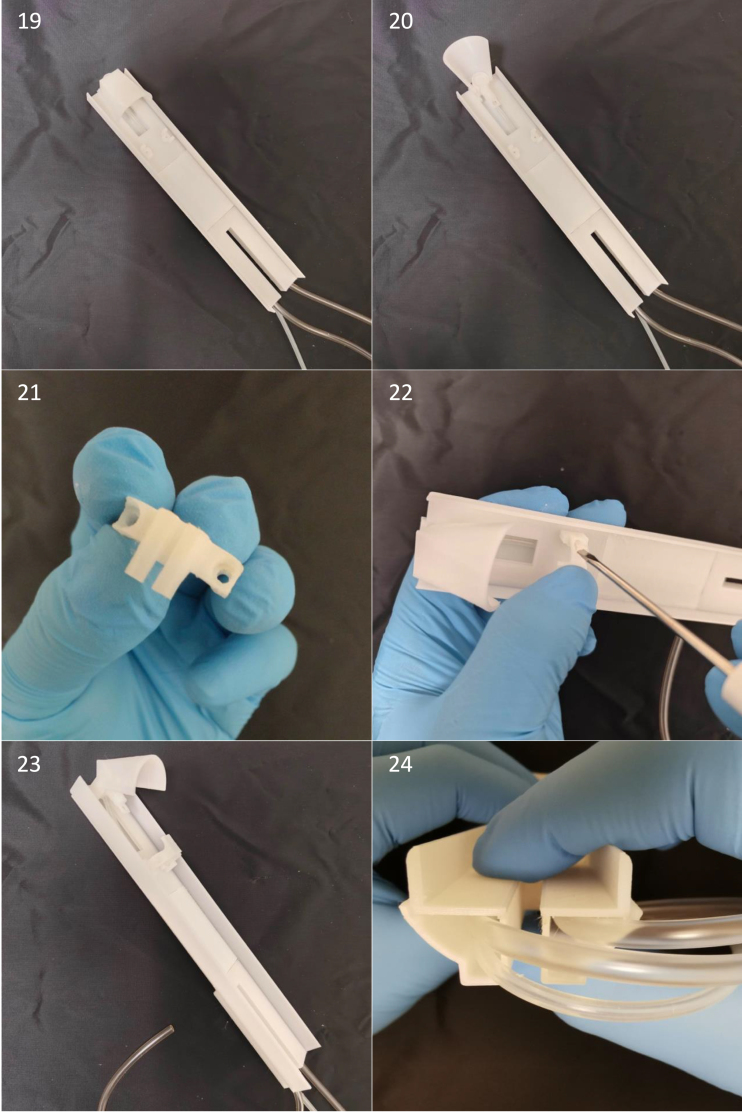
Step 19–20: View of the mouse cradle with the hinging anaesthesia mask. Step 21–23: The mouse head collar has two holes on either sides. place the head collar down to the mouse cradle’s head stage and screw it down to it by using two M2 nylon bolts. Step 24: Notice here, due to the space restriction the two water tubes for water container need to be on each of the two small cavities of the mouse cradle (rear bottom).

The build instructions for the mouse cradle are aided by pictures of its components and construction (see Figs. 12 and Fig. 14, Fig. 15, Fig. 16, Fig. 17). This cradle is made for both awake and anaesthetized scans. When working with anaesthetized animals, body temperature needs to be externally regulated. Commercially available solution to this (Fig. 13(a)) has a couple of limitations, 1: the blue pad is placed on top of the mouse’s back. This is ineffective given that the mouse’s thermal window is at the underbelly, 2: Given that water flows in the direction with the least resistance, the black hollow box connecting the tubes for water in and outflow is situated before the blue pad with the risk of water shunting with little water flow through the blue pad (see green arrows Fig. 13(a)). Our setup has warm water flow integrated in the cradle bed floor. Thermal regulation is therefore done in direct contact with the mouse’s underbelly, with direct water in- and outflow ensuring stable water circulation (see Fig. 13(b)).

## 5 Build and operation instructions

### Assembly of suspension system

**Fig. 18:**
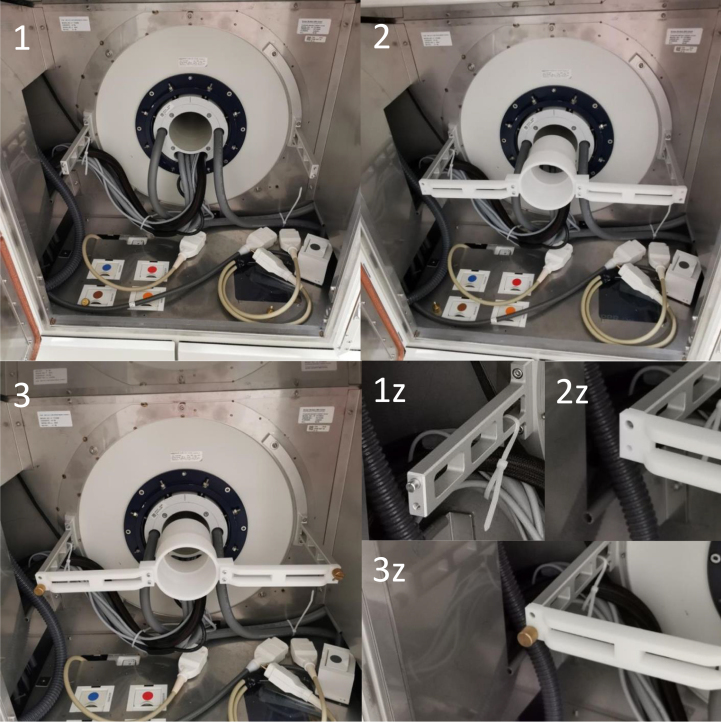
Assembly steps of the rear attachment site for the suspension system.

**Fig. 19:**
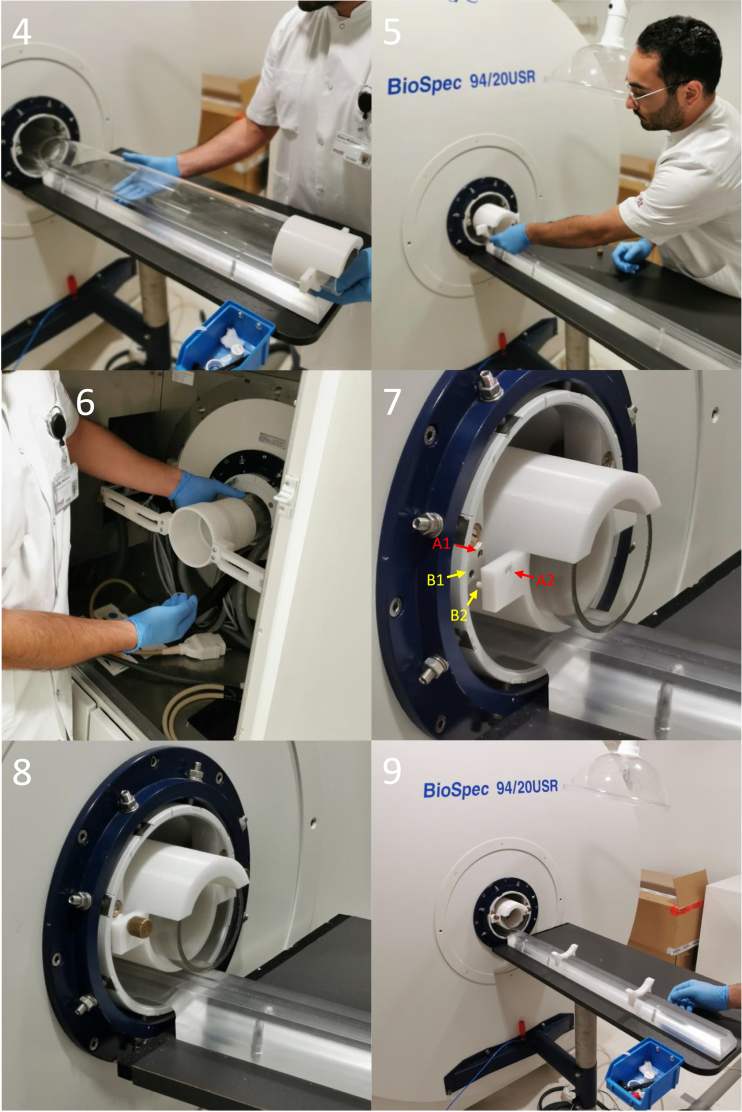
Assembly steps of the front attachment site for the suspension system.

Fig. 18 step 1 and 1z illustrates Bruker’s rear attachment site with no rear acrylic tube mount installed. At the rear attachment site, the rear acrylic tube mount is installed (see Fig. 18 step 2 and 2z). Using a brass bolt, the rear acrylic tube mount is fixed to the rear attachment site (see Fig. 18 step 3 and 3z). Next, the acrylic tube with the front acrylic tube mount attached is inserted into the scanner bore (see Fig. 19 step 4–5). It is then guided further into the rear acrylic tube mount (see Fig. 19 step 6). Afterwards, the front acrylic tube peg (B2) is inserted into peg holes in Bruker’s front attachment site (B1) (see Fig. 19 step 7). Finally, the front acrylic tube mount is fixed to the front attachment site with a brass bolt (A1 and A2) (see Fig. 19 step 8–9).

### Assembly and operation for the ${}^{1}$H/^31^ P volume coil

**Fig. 20:**
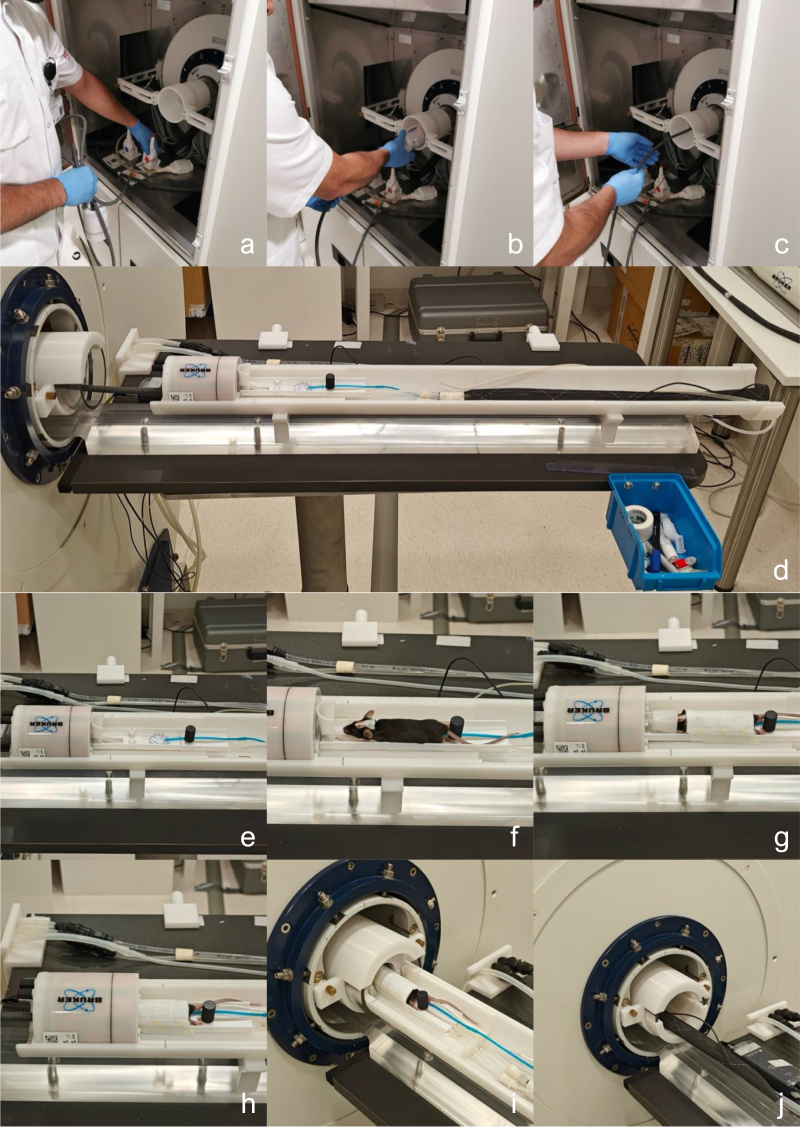
Illustration of the setup of the ${}^{1}$H/^31^P volume coil, including its connection to the scanner, operation of the rails and the placement of an anaesthetized mouse on the mouse cradle.

The ${}^{1}$H/^31^P volume coil’s ${}^{1}$H and ^31^P channels are connected to their colour-coded ${}^{1}$H and ^31^P sockets. The coil is then gently guided through the acrylic tube (see Fig. 20a–c). The inner and outer rails are assembled in front of the scanner bore. The volume coil is placed into the inner rail’s coil bed. The inner rail has, in front of the volume coil bed, a mouse cradle rail, where our custom mouse cradle is placed. The rail allows the mouse cradle to slide back and forth. The mouse cradle rail has two holes with M5 threads, allowing the mouse cradle to be fixed at any position using an M5 nylon bolt (see the black cylinder shaped bolt head in Fig. 20d). The mouse cradle is attached to a water inflow and outflow system. The mouse cradle also allows monitors for respiration rate to be placed on the bed plate (round pad with blue tube in Fig. 20d–e). An anaesthetized mouse is placed on the mouse cradle bed, and its head collar is screwed to the mouse cradle’s head stage using an M2 nylon bolt on each side (see Fig. 20e–f). An anaesthesia tube is positioned in front of the mouse, with the mouse’s front teeth placed in the anaesthesia tube’s tip. The anaesthesia mask is tilted down to encapsulate the gases, and a piece of tissue with tape fixes the mouse’s body to the mouse cradle (see Fig. 20e–g). From here, an anaesthetized scan can be performed by inserting a rectal probe to monitor and control body temperature by regulating the water temperature that flows into the cradle. For awake scans, the anaesthesia and water flow is shut off, and the anaesthesia tube is moved forward away from the mouse, allowing it to wake up. The volume coil’s isocenter is indicated by the black line. The organ of interest is placed at the isocenter of the volume coil by sliding the mouse cradle forward and fixing it with the M5 nylon bolt (see Fig. 20g–h). The entire rail system is inserted into the acrylic tube. The outer rail has a socket at the end that snugly clamps to the acrylic tube, ensuring the rails to be fixed to the acrylic tube (see Fig. 20h–j).

### Assembly and operation for the ${}^{1}$H/^31^ P surface coil

**Fig. 21:**
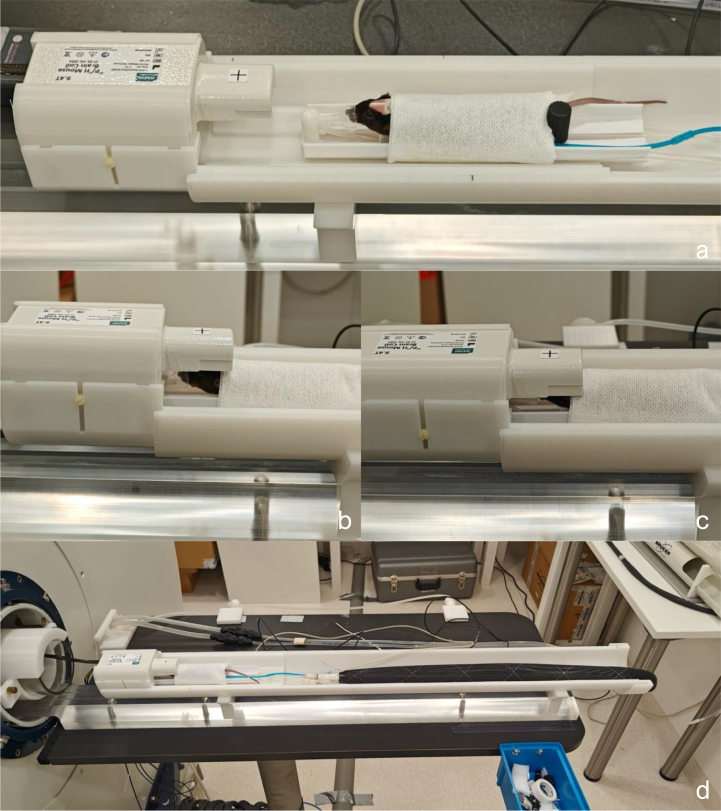
Illustration of the setup of the ${}^{1}$H/^31^P surface coil, including operation of the rails and the placement of an anaesthetized mouse on the mouse cradle.

Similar to the volume coil, the ${}^{1}$H/^31^P surface coil’s ${}^{1}$H and ^31^P channels are connected to the colour-coded scanner sockets, and the surface coil is guided through the suspension system’s acrylic tube. The surface coil is placed into its dedicated socket and the socket into the rail (see supplementary fig. 23 f–h). The same mouse cradle for the volume coil is used, with the mouse positioned the same way on the mouse cradle rail in front of the coil. However, the anaesthesia mask gets in the way of the surface coil, instead a mask made from a cut-open pipette bulb is used to encapsulate the gases coming from the anaesthesia tube (see Fig. 21a). The surface coil’s isocenter is marked by a cross. The mouse cradle is moved forward such that the mouse’s brain is positioned below the surface coil’s isocenter. The mouse cradle is then fixed to the mouse cradle rail using an M5 nylon bolt (see Fig. 21a–b). The surface coil is then lowered onto the mouse’s head, and its position is secured using the M2 nylon bolts on the sides (see Fig. 21b–c). Finally, the entire rail setup for the surface coil is ready to be inserted into the acrylic tube, as done with the volume coil (see Fig. 21d).

## 6 Validation and characterization

**Fig. 22:**
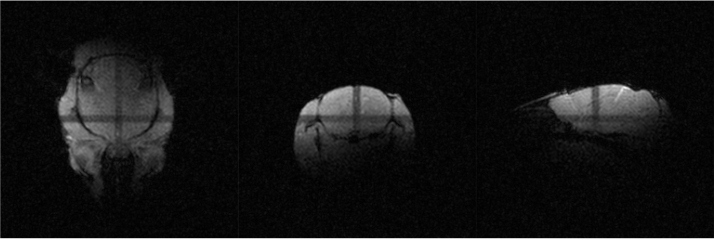
Localized image with an axial, coronal and sagittal orientation.

The MCSS needs to be compatible with the common MRS workflow, which requires the ability to position the coil centre, with the organ of interest in it, at the scanner’s isocenter. The inner rail’s position on the outer rail can be adjusted to place both the coils and the organ of interest at the scanner’s isocenter. This positioning only needs to be done once, with the position marked. Fig. 22 illustrates the first localizer scan (scout) performed after inserting an awake mouse into the system using the surface coil. During anaesthetized scans, air suction is placed next to the scanner front bore to prevent the accumulation of anaesthesia and the potential deterioration of the acrylic tube due to its exposure over time.

## CRediT authorship contribution statement

**Saba Molhemi:** Writing – review & editing, Writing – original draft, Visualization, Validation, Project administration, Methodology, Investigation, Conceptualization. **Leif Østergaard:** Funding acquisition. **Brian Hansen:** Writing – review & editing, Visualization, Validation, Supervision, Resources, Investigation, Conceptualization.

## Ethics statements

All procedures were carried out with approved permits from the Danish Animal Veterinary Inspectorate (permit number 2020-15-0201-00684).

## Funding

This work was supported by Prof. Leif Østergaard’s grant from the 10.13039/501100003554Lundbeck Foundation, Denmark (R310-2018-3455).

## Declaration of competing interest

The authors declare that they have no known competing financial interests or personal relationships that could have appeared to influence the work reported in this paper.

## Appendix A Supplementary data

MMC S1
