# Supplementary material for: A low-cost open-source 3D-printed mouse cradle suspension system for awake or anaesthetised 1H/31P magnetic resonance spectroscopy
Source: HardwareX. 2024 Dec 30;21:e00616. doi: 10.1016/j.ohx.2024.e00616 (PMC11783022; doi:10.1016/j.ohx.2024.e00616)
Supplement: MMC S1 [file mmc1.pdf]

7. Supplementary material

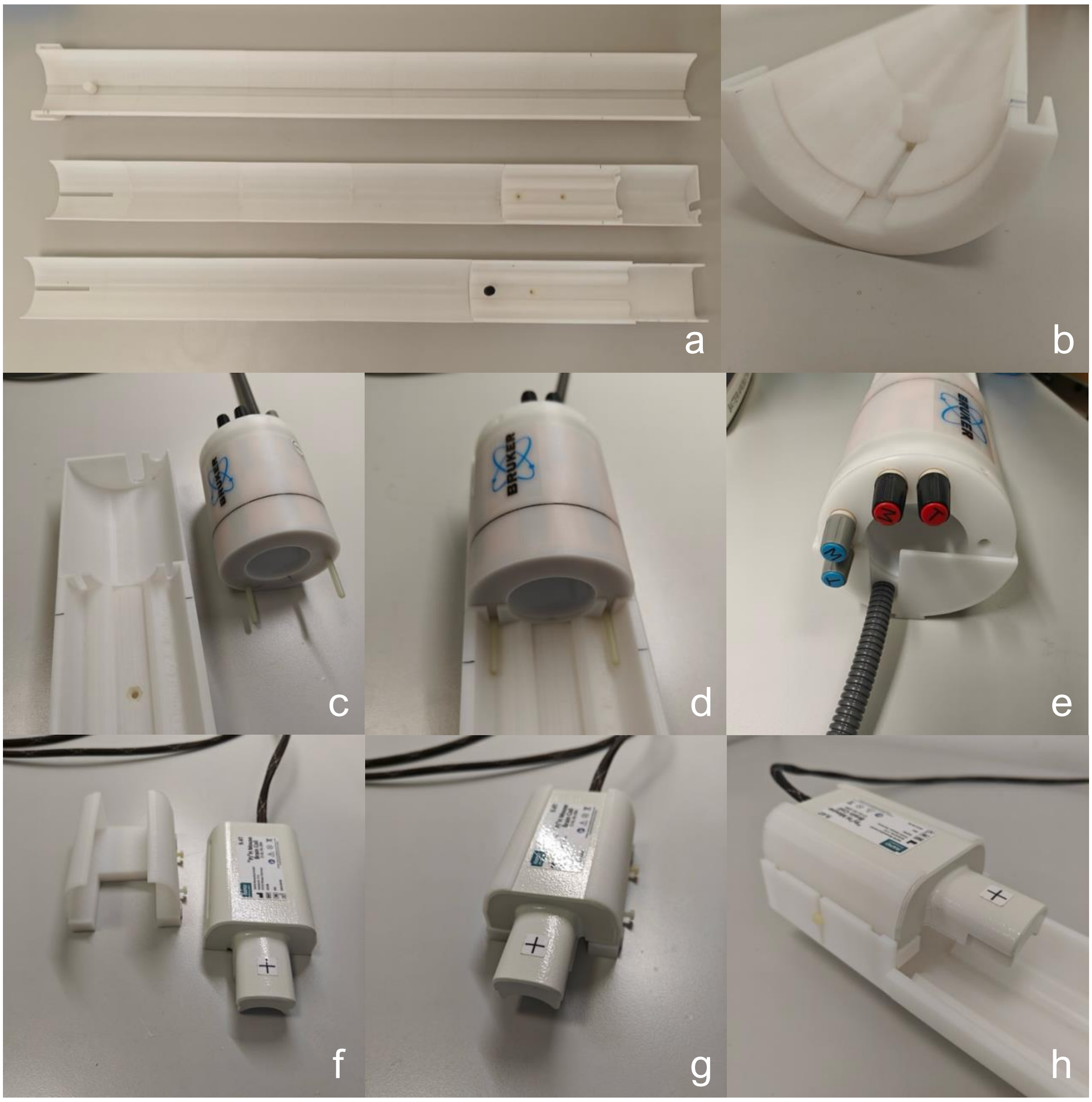

Figure 23: Image a: The outer rail at the top, the inner rail (volume coil) in the middle, and the inner rail (surface coil) at the bottom. Image b: Both inner rails have a gap in the front end, and the outer rail has a M5 thread at its front end. This allows the inner rails to slide into the outer rail and be fixed by using a M5 nylon bolt. Images c–e: The volume coil is snugly placed into the volume coil bed. Both inner rails have in front of their coils a mouse cradle rail. Image f–h: The surface coil is positioned into the surface coil socket, and then positioned into the surface coil rail.

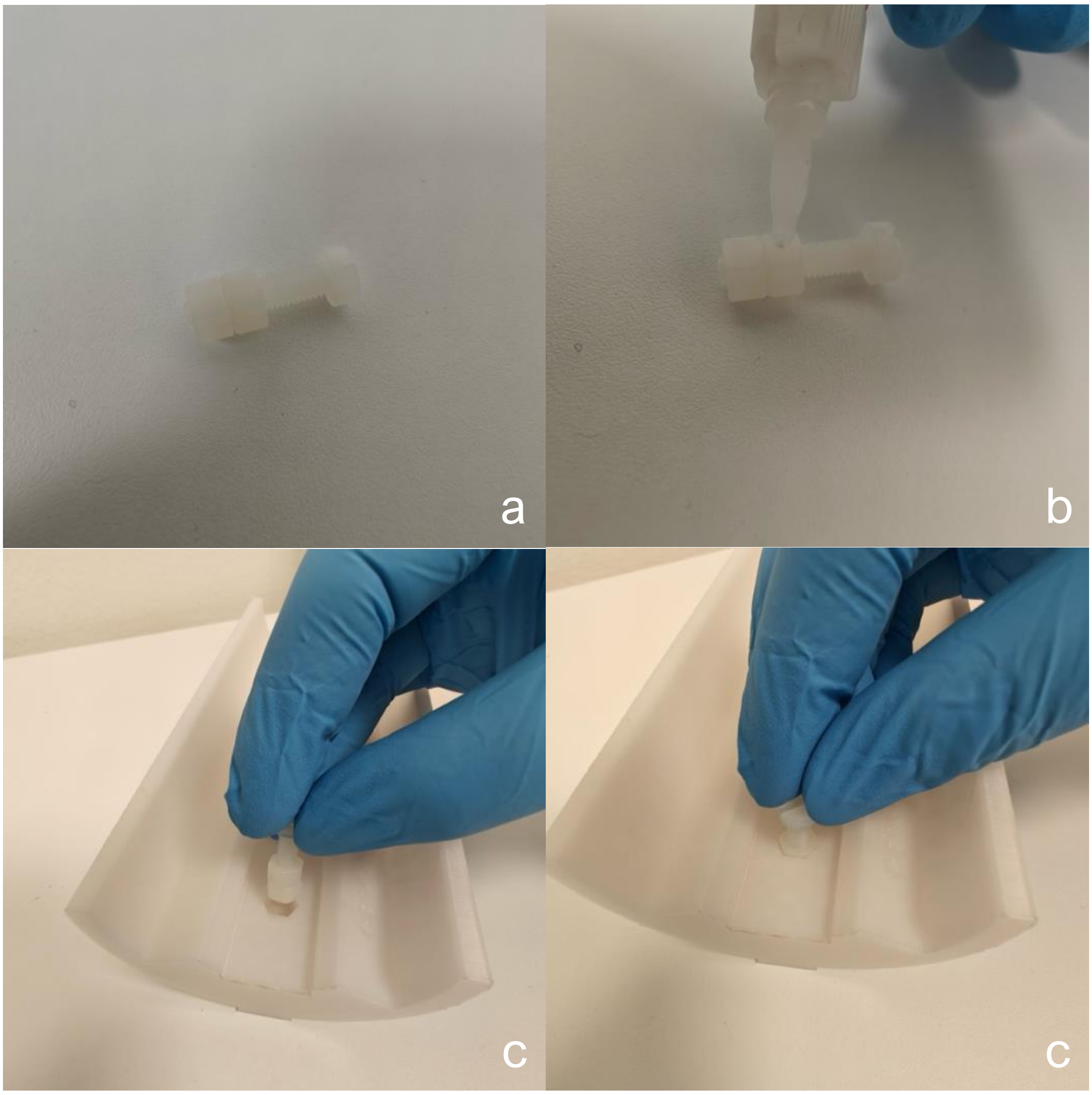

Figure 24: Image a: Two M5 nylon nuts are screwed onto the tip of an M5 nylon bolt. The nuts need to be next to each other and aligned. Image b: Loctite super glue power gel is applied to the sides of both nuts. Image c–b: The M5 nylon bolt with the two nuts at the end is inserted into the hexagon-shaped hole in the mouse cradle rail so that the nuts are flush with the surface. After letting the glue dry, the M5 nylon bolt is unscrewed, leaving the two M5 nylon nuts in the hole.

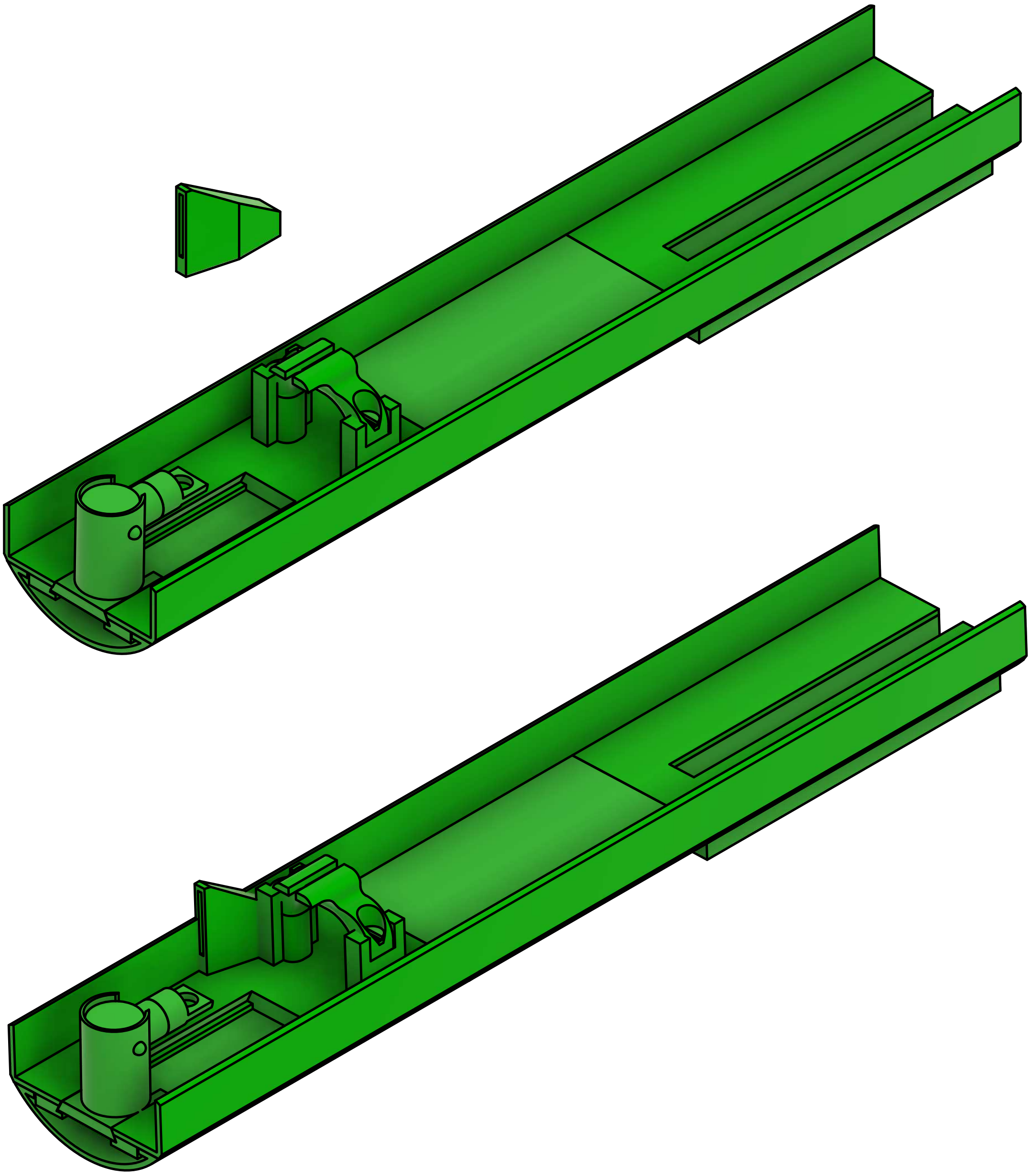

Figure 25: Whisker stimulation tube wedged into position between the bed side-wall and head-stage on the mouse cradle.
